# On Conducting a Medical Education

**Published:** 1818-03

**Authors:** 


*For the London Medical and Physical Journal.*

*On Conducting a Medical Education.*

IN answer to the enquiry of Pater-familias, respecting the best mode of conducting the academic education of his son at the Edinburgh University, I beg leave to offer a few hints; and, as I was for some years *alumnus* of that and another celebrated northern university, I may be presumed to be not altogether incompetent to give an opinion on the subject.

Every candidate for the degree of M.D. at the Edinburgh University, must have attended a certain number of medical lectures, during a residence of at least three winters and one summer, either at that or some other university. Before one can be admitted as a candidate for a degree in medicine at the Glasgow University, he must have resided there during at least one session; but the stated time of residence and attendance on lectures at *any* regular university, I believe, entitles one to an examination at the Edinburgh. The regulations of these two universities, respecting medical degrees, are in other respects similar, except that, at the Glasgow, an attendance on one course of midwifery is required, which is not the case at the Edinburgh; and, at the former, one may obtain a degree without publishing a thesis, which is never dispensed with at the latter. The session lasts the winter's six months; the summer's residence, required of candidates for a medical degree, is for the purpose of learning botany.

Though the Edinburgh University requires only a three winters' residence of candidates for a medical degree, I am one of those who think that time too short for accomplishing the academic education of a physician; and I would strongly recommend Juvenis, provided his arrangements may permit, to study, at least, four sessions. By doing so, he will not only have an opportunity of studying more fully the various branches of his profession, but also, (without infringing too much on the time necessary for these) of attending lectures on a few collateral sciences, such as logic, natural and moral philosophy, and natural history; some knowledge of which, though, perhaps, not essential, it will be admitted, is ornamental and useful to a physician, and may have a favourable influence on his success in life. That a physician, besides his professional knowledge, should have an education in every respect liberal, and, particularly, as much knowledge as possible of the mental operations, is a proposition which, I believe, will be assented to by those who are best qualified to judge on the subject.—"Etenim omnes artes quæ ad humanitatem pertinent habent quoddam commune vinculum et suasi cognatione quadam inter se continentur." And, I may add, that I have heard judicious physicians, educated at the northern universities, regret that they had not, during their residence there, attended lectures on the above sciences, as a favourable opportunity for that purpose had never afterwards occurred to them. At the English universities, no medical students, but those who are Bachelors of Arts, are admitted as candidates for a degree in medicine; whilst, at the northern universities, a previous attendance on medical lectures only entitles one to an examination; though, I think it would add to the respectability of these latter, if they were to be more particular respecting the general education of those whom they admit to the highest honors of medicine. It may, indeed, be objected, that the number of medical lectures to be attended during the three winters' residence at the northern universities, is amply sufficient to exercise the industry of young men, particularly of those who resort thither without any previous initiation in the profession; but, this objection would be obviated, if a residence of four, instead of three, sessions should be required of candidates for medical degrees. Your correspondent's son, from having served an apprenticeship and walked the hospitals in London, will certainly be better able than many young men who repair to the university without such initiatory education, to dispense with a longer residence at it than that required.—Still, however, I can assure him, that he may study four sessions at Edinburgh with the greatest advantage; and that, when he is once embarked in the arduous duties of his profession, he will not think too long any time which he has passed in qualifying himself for their successful and conscientious discharge.

In the commencement of his studies at the university, the judgment of Juvenis will be exercised on the subject of taking notes during the lectures. If a good stenographist, he will not be easily persuaded from writing during the lectures, though the practice, I conceive, is not generally advisable, as it has a tendency to distract the intellectual powers, weaken the memory, and prevent that attention to a professor's countenance, from which an intelligent student may often derive no inconsiderable assistance, as, I believe, will be assented to by those who have had the good fortune to be pupils of the present distinguished professor of the practice at Edinburgh. Besides, as notes taken during the lectures are generally scrawled in inelegant characters, and the decyphering of them of course is irksome; and as the student will, in the course of his education, find that there is but little information in them which may not be gained from reading, it frequently happens that he neglects, and never even once peruses, them. I should recommend a student to abstain from writing during lectures; but, unless his memory should be very tenacious, on his return to his lodging, to set down whatever he may think most interesting, or worthy to be remembered. This plan, at the same time, excites reflection on medical subjects, and exercises and strengthens the memory.

There is, attached to the university of Edinburgh, an excellent library, the use of which students have by paying a small annual subscription; and, as it is well supplied with books in every branch of medicine, I would recommend Juvenis to become a subscriber. I shall take the liberty of mentioning a few books, which I think may be peculiarly deserving of his attention. On chemistry, there is, perhaps, no elementary work better calculated to give one a taste for that very interesting science than Park's Chemical Catechism; and, as a more comprehensive work on the same subject, I know none preferable to Dr. Thompson's, or Mr. Murray's, System. On Materia Medica and Pharmacy, I should recommend Mr. Murray's System of Mat. Med. and Phar., the Apparatus Medicaminum of Professor Murray, of Gottingen; and the Edinburgh New Dispensatory. To enumerate even the names of all the works which may be perused with advantage on the Practice of Medicine, would occupy more space than is allotted to this paper. The following, however, are a few of those which I conceive are peculiarly excellent:—Cullen's First Lines; the Edinburgh Practice of Physic; Thomas's Practice of Physic; Edinburgh Medical Essays; Adams on Morbid Poisons; Darwin's Zoonomia, for the practical information it communicates; Bree on Disordered Respiration; Hamilton on Purgatives; Watts on Diabetes; Pemberton on Diseases of the Abdominal Viscera; Saunders on the Liver; and Young's Medical Literature. To students who had a prospect of practising in the army, or in warm climates, I would recommend Pringle on the Diseases of the Army; Cleghorn on the Diseases of Minorca; Irwine on the Diseases of Sicily; but, above all, the publications of Dr. Robert Jackson,—perhaps, the most experienced army-physician that any age or country ever produced. Haller's Primæ Liniæ, and Gregory's Conspectus Medicinæ Theoreticæ, are, in my opinion, the two best works on Physiology. Anatomy is to be properly studied in the Book of Nature only; but, good works on the subject, such as those of Monro, Fyfe, Bell, &c., with correct drawings, will render the student considerable assistance. The best elementary work on Surgery, is, perhaps, Cooper's First Lines; and, to those who have made some progress in that science, I should recommend an attentive perusal of the writings of Sharp, Bloomfield, Pott, John Hunter, Scarpa, Richter, Abernethy, Carmichael, Hey, Larrey, &c. Those who read French with facility, can scarcely perhaps be too conversant with the Memoir. de l'Academ. de Chirurg.,—a noble monument of French talents and industry, and one to which surgeons will, in every succeeding age, frequently refer. In this concise and imperfect enumeration of distinguished authors on the different branches of medicine, I have omitted the ancients, as I conceive, that, during an academic education, is not the most proper time for consulting them. I have, also, omitted the periodical works on medicine, though, with the venerable Rush, I conceive, that the faculty are deeply indebted to many of them for promoting the march of professional knowledge, and to none so much for contributing to this desirable end as to the London Medical and Physical Journal.

No books are used in the examinations for a medical degree at Edinburgh, though there are some, which I conceive may be previously read by the candidate with much advantage,—such as Celsus, the Latin writings of Harvey, Sydenham, Boerhaave, and Haller; Brown's Elementa Medicinæ, and Gregory's Conspectus Medicinæ Theoreticæ; as, from a perusal of these, he is likely to improve himself in the knowledge of Latin, and of his profession at the same time. The candidate has, besides undergoing two or three private examinations by some of the professors, to publish a Thesis Inauguralis, and defend it *coram pleno senatu*. The Thesis is written in Latin; and, as the subject of it is optional to the candidate, he generally selects some disease or function which he has closely studied, and which he may suppose himself peculiarly competent to illustrate. The private examinations, with the public defence of his Thesis, are also carried on in Latin; so that it is incumbent on him to have a tolerable knowledge of that language; for, though classical elegance is not generally expected, it is supposed that, having received a liberal education, he shall be able to acquit himself in a respectable manner. Your Correspondent's son is stated to have received a good classical education; this, I can assure him, will prove useful to him, not only in writing his Thesis, and in his examinations, but at every step of his professional education. Every one acquainted with the Scottish universities knows, that it is possible for a student, who has gone through the usual routine of medical education at them, though he is but little versed in the Latin language, to obtain a degree by the aid of the grinders, as they are called; but, to be indebted to those *dii e machina* for obtaining the honours of his profession, must, I should think, be to a gentleman, through life, an humiliating reflexion.

As the candidate has attended lectures on the different branches of the profession, and is, of course, supposed to have a competent knowledge of them all, the professors in the private examinations, *per universam eum medicinam curant examinare.* But, though he may be interrogated *per universam medicinam*, the questions, I believe, principally relate to the anatomy of the viscera, the practice of medicine, and the Matera Medica. If the candidate has acquitted himself to the satisfaction of the professors in these examinations, and in the writing of his Thesis, a day is appointed for him to defend it publicly, when the degree of M.D., with the usual ceremonies, is conferred on him.

I would recommend Juvenis, particularly should he determine on a four-winters' residence in Scotland, previously to graduation, to pass the second session at the Glasgow university, where there is, as at Edinburgh, a complete medical school, conducted by gentlemen whose learning, talents, and experience, entitle them to high consideration. Two of them, Dr. Watt and Mr. Burns, are well known to the medical world by their writings, and I know others of them want nothing but the inclination to be equally distinguished as authors. Juvenis would, from a winter's residence at the university, not only have an opportunity of comparing the doctrines of its able professors with those of the Edinburgh school, but also of repeatedly examining the invaluable preparations illustrative of every branch of medicine, in the late Dr. William Hunter's extensive museum, now belonging to the University of Glasgow.

During the two years which he proposes to reside in London, previously to his repairing to the university, I should recommend him, besides maintaining his correspondence with his old friends Virgil, Horace, Homer, &c. to attend lectures on Natural Philosophy; and to acquire, if he have not already done so, some knowledge of the Mathematics.

Should there be any point on which your correspondent, Pater-familias, is desirous of more particular information, I shall be happy, if it be in my power, to give it him through the medium of your excellent Journal.

*Northampton; Jan.* 29, 1818. L.

---

*For the London Medical and Physical Journal.*

